# Loganin Alleviates Gout Inflammation by Suppressing NLRP3 Inflammasome Activation and Mitochondrial Damage

**DOI:** 10.3390/molecules26041071

**Published:** 2021-02-18

**Authors:** Nuri Choi, Gabsik Yang, Joo Hyeon Jang, Han Chang Kang, Yong-Yeon Cho, Hye Suk Lee, Joo Young Lee

**Affiliations:** 1 BK21plus Team, College of Pharmacy, The Catholic University of Korea, Bucheon 14662, Korea; snfzk@catholic.ac.kr (N.C.); jangzoo1140@naver.com (J.H.J.); hckang@catholic.ac.kr (H.C.K.); yongyeon@catholic.ac.kr (Y.-Y.C.); sianalee@catholic.ac.kr (H.S.L.); 2 Department of Pharmacology, College of Korean Medicine, Woosuk University, Jeonbuk 553382, Korea; yanggs@woosuk.ac.kr

**Keywords:** innate immunity, inflammation, pharmacological inhibitor, mitochondria, cytokine

## Abstract

Gout is a type of inflammatory arthritis caused by the deposition of monosodium uric acid (MSU) crystals in tissues. The etiology of gout is directly linked to the NLRP3 inflammasome, since MSU crystals are NLRP3 inflammasome activators. Therefore, we decided to search for a small-molecule inhibitor of the NLRP3 inflammasome for the prevention of gout inflammation. We found that loganin suppressed MSU crystals-induced caspase-1 (p20) and interleukin (IL)-1β production and apoptosis-associated speck-like protein containing a caspase recruitment domain (ASC) specks formation in mouse primary macrophages, showing its ability to inhibit the NLRP3 inflammasome. In an air pouch inflammation model, oral administration of loganin to mice prevented MSU crystals-induced production of mature IL-1β and IL-18 in air pouch exudates, resulting in decreased neutrophil recruitment. Furthermore, oral administration of loganin suppressed MSU crystals-induced gout inflammation in a mouse foot gout model, which was accompanied by the inhibition of the NLRP3 inflammasome. Loganin blocked de novo synthesis of mitochondrial DNA in air pouches and foot tissues injected with MSU crystals. Consistently, loganin prevented MSU crystals-induced mitochondrial damage in macrophages, as it increased mitochondrial membrane potential and decreased the amount of mitochondrial reactive oxygen species. These data demonstrate that loganin suppresses NLRP3 inflammasome activation by inhibiting mitochondrial stress. These results suggest a novel pharmacological strategy to prevent gout inflammation by blocking NLRP3 inflammasome activation and mitochondrial dysfunction.

## 1. Introduction

The inflammasome is one of the pattern recognition receptors responding to pathogen-associated molecular patterns derived from invading pathogens [1,2]. The inflammasome is a multi-protein complex that processes the cleavage of pro-interleukin (IL)-1β and pro-IL-18 to mature IL-1β and IL-18 and triggers inflammatory cell death, i.e., pyroptosis [3]. The NLRP3 (NOD-, LRR-, and pyrin domain-containing protein 3) inflammasome consists of NLRP3, apoptosis-associated speck-like protein containing a caspase recruitment domain (ASC), and pro-caspase-1. NLRP3, acting as a sensor, is composed of an amino-terminal pyrin domain (PYD), a NACHT domain, and a carboxy-terminal leucine-rich repeat (LRR) domain. ASC is composed of an amino-terminal PYD and a carboxy-terminal caspase recruitment domain (CARD). Caspase-1 has an amino-terminal CARD consisting of the catalytic domains p20 and p10. NLRP3 interacts with ASC through PYD–PYD association, while the interaction between ASC and pro-caspase-1 takes place via CARD–CARD interaction [4,5]. Activation of caspase-1 is achieved by proximity-induced autocatalytic activation, leading to the cleavage of the pro-forms of IL-1β and IL-18 to biologically active forms [6]. In addition, active caspase-1 induces the degradation of gasdermin D (GSDMD) to form pores consisting of the N-terminal domain of GSDMD in the plasma membrane, thereby triggering pyroptotic cell death [7]. The NLRP3 inflammasome is also activated by damage-associated molecular patterns including ATP, K^+^ ionophores, bacterial toxins, and particulate matters, in addition to infectious bacteria [8,9]. The activation of the NLRP3 inflammasome plays a critical role in the regulation of host immune responses and is closely linked to the pathogenesis of inflammatory diseases such as autoinflammatory diseases, atherosclerosis, non-alcoholic fatty liver disease, diabetes, and gout [10]. Therefore, the pharmacological inhibition of NLRP3 activation is thought to exert potent therapeutic effects on a wide variety of inflammatory diseases [11].

Gout is a painful type of inflammatory arthritis, whose incidence has been increasing in modern society due to changes in human living conditions, lifestyle, and eating habits [12]. The medical treatment of gout includes nonsteroidal anti-inflammatory drugs (NSAIDs), steroids, colchicine, and uric acid-lowering medications. Gout patients may experience relapsing gout flares resulting in the erosion and destruction of a joint. Gout is triggered by the accumulation of monosodium urate (MSU) crystals, the final metabolite of purines, in the big toe or other joints, causing inflammatory arthritis with swelling and intense pain, called gout attacks. It is now well established that MSU crystals induce the activation of the NLRP3 inflammasome, resulting in the production of pro-inflammatory cytokines such as IL-1β and IL-18 and subsequent inflammatory cell death [13]. Therefore, the NLRP3 inflammasome could be a potential target for gout therapy.

**Figure 1:**
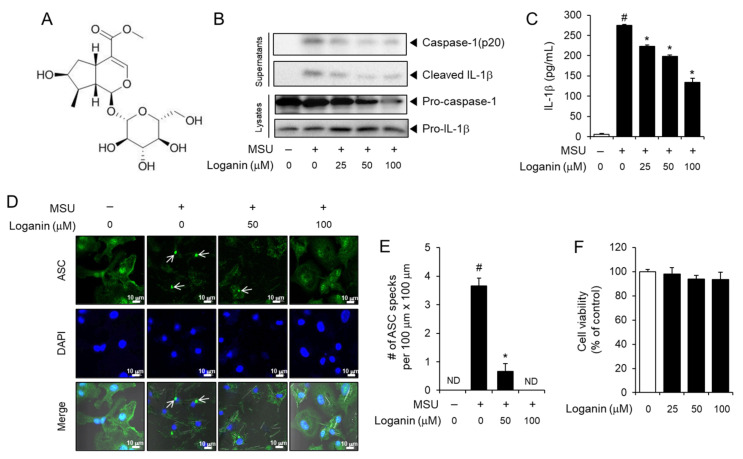
Loganin inhibits monosodium urate (MSU) crystals-induced activation of the NLRP3 inflammasome in primary macrophages. (**A**) Chemical structure of loganin. (**B**–**D**) Bone marrow-derived mouse primary macrophages (BMDMs) were primed with lipopolysaccharide (LPS) (100 ng/mL) for 4 h. The cells were treated with loganin for 1 h and then stimulated with MSU crystals (500 μg/mL) for (**B**) 6 h or (**C**–**E**) 4.5 h. (**B**) Cell culture supernatants and cell lysates were analyzed by immunoblotting for pro-caspase-1, caspase-1(p20), pro-interleukin (IL)-1β, and mature IL-1β. (**C**) Cell culture supernatants were analyzed for secreted IL-1β by ELISA. (**D**) BMDMs were fixed, permeabilized, and stained for apoptosis-associated speck-like protein containing a caspase recruitment domain (ASC, green), and the nuclei were stained with 4′,6-diamidino-2-phenylindole (DAPI, blue). The arrows indicate ASC specks. Scale bars = 10 µm. (**E**) The bar graph represents the number of ASC specks per 100 μm × 100 μm, which was obtained from different fields of view (*n* = 3). (**F**) Cell viability of BMDMs was determined by the MTT assay after the cells were treated with loganin at the indicated concentrations for 7 h. The values in the bar graphs represent the means ± SEM (*n* = 3); # significantly different from vehicle alone, *p* < 0.05; * significantly different from MSU alone, *p* < 0.05; ND, not detected.

We intended to search and develop a new pharmacological inhibitor of the NLRP3 inflammasome for the prevention of gout inflammation. Loganin is an iridoid glycoside compound (Figure 1A) with anti-inflammatory activity [14]. However, it has not been examined whether loganin can regulate the activation of the NLRP3 inflammasome and be effective to treat gout. Therefore, we investigated whether loganin has suppressive effects on the NLRP3 inflammasome activation induced by MSU crystals and may consequently prevent gout inflammation in mouse primary macrophages and mouse acute gout models.

## 2. Results

### 2.1. Loganin Suppresses MSU Crystals-Induced Activation of the NLRP3 Inflammasome in Primary Macrophages

To investigate whether loganin inhibited MSU crystals-induced activation of the NLRP3 inflammasome, bone marrow-derived primary mouse macrophages (BMDMs) were treated with loganin after the cells were primed with lipopolysaccharide (LPS) and further stimulated with the MSU crystals. Loganin treatment prevented the cleavage of pro-caspase-1 to caspase-1 (p20) and the degradation of pro-IL-1β to mature IL-1β induced by the MSU crystals in primary mouse macrophages, as determined by immunoblot analysis (Figure 1B). Similarly, loganin decreased MSU crystals-induced secretion of mature IL-1β in cell culture supernatants, as determined by ELISA (Figure 1C). The formation of ASC specks is another indicator of NLRP3 inflammasome activation. Confocal imaging analysis showed that loganin suppressed MSU crystals-induced formation of ASC specks, confirming its inhibitory effect on NLRP3 inflammasome activation (Figure 1D,E). We determined the effect of loganin on cell viability by the MTT assay. Loganin within the concentration ranges used in the experiment for 7 h did not reduce the viability of BMDMs (Figure 1F). Loganin did not exhibit inhibitory effects on the production of IL-6 and tumor necrosis factor (TNF)-α, whose expression is independent of NLRP3 inflammasome activation (Figure S1A,B). These results indicate that loganin suppresses MSU crystals-induced activation of the NLRP3 inflammasome in macrophages.

### 2.2. Oral Administration of Loganin Suppresses MSU Crystals-Induced NLRP3 Inflammasome Activation in Mice

**Figure 2:**
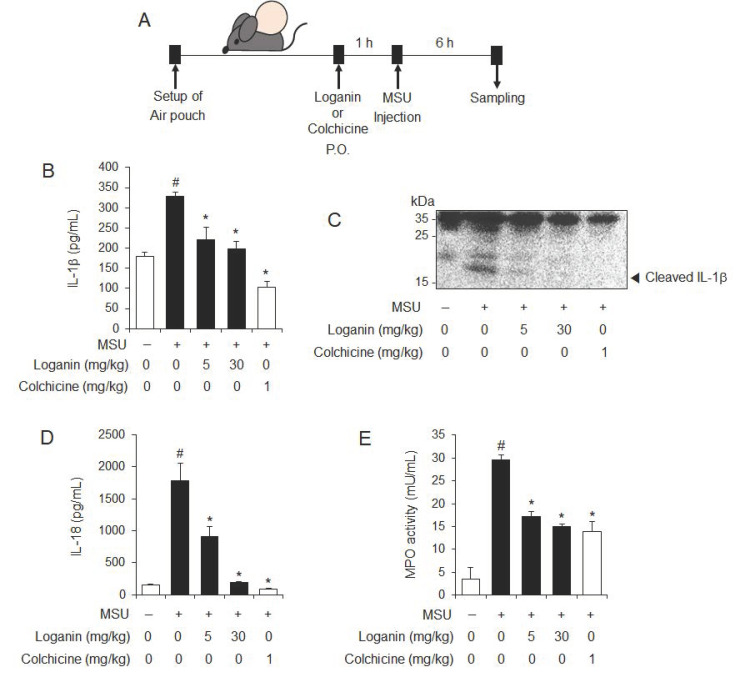
Oral administration of loganin inhibits MSU crystals-induced NLRP3 inflammasome activation and inflammation in a mouse air pouch model. Air pouches were formed on the mouse back by injecting air twice. Loganin (5 or 30 mg/kg) or colchicine (1 mg/kg) was orally administered. After 1 h, MSU crystals (3 mg/mL in PBS/mouse) or PBS alone were injected into the air pouches. After 6 h, the pouch exudates were harvested and the supernatants were analyzed. (**A**) Experimental scheme. (**B**) ELISA for IL-1β. (**C**) Immunoblotting for IL-1β. (**D**) ELISA for IL-18. (**E**) Myeloperoxidase (MPO) activity, which reflects neutrophil recruitment, was assessed in the air pouch exudates. The values in the bar graphs represent the means ± SEM (*n* = 5 mice); # significantly different from vehicle alone, *p* < 0.05; * significantly different from MSU alone, *p* < 0.05.

We next investigated whether loganin had an in vivo inhibitory effect on NLRP3 inflammasome activation using a mouse air pouch model. After air pouches were formed on the backs of mice, the mice were orally administered 5 or 30 mg/kg of loganin (Figure 2A). Colchicine was used as a positive control, since it is a medication used to treat gout. One hour later, MSU crystals were injected into the air pouch to activate the NLRP3 inflammasome (Figure 2A). Oral administration of loganin reduced mature IL-1β production in air pouch exudates, which was increased by MSU crystals injection, as shown by ELISA and immunoblotting (Figure 2B,C). In addition, loganin reduced MSU crystals-induced IL-18 production, whose cleavage from pro-IL-18 is mediated by NLRP3 inflammasome activation (Figure 2D). Furthermore, oral administration of loganin suppressed myeloperoxidase (MPO) activity in the air pouch, which was increased by MSU crystals injection, suggesting that loganin blocked inflammatory responses induced by NLRP3 inflammasome activation (Figure 2E). The suppressive effects of loganin on the production of IL-1β and IL-18 and the MPO activity in air pouch exudates were similar in extent to those of colchicine. These results demonstrate in vivo suppressive effects of loganin on NLRP3 inflammasome activation induced by MSU crystals, which were well correlated with inhibition of inflammation.

### 2.3. Oral Administration of Loganin Suppresses MSU Crystals-Induced Gout Inflammation in a Mouse Foot Gout Model

**Figure 3:**
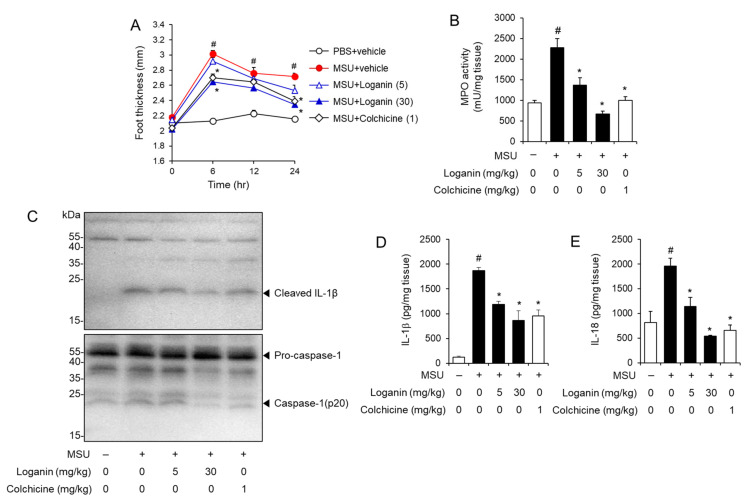
Oral administration of loganin alleviates MSU crystals-induced inflammatory symptoms and NLRP3 inflammasome activation in a mouse acute gout model. Mice were orally administered loganin (5 or 30 mg/kg) or colchicine (1 mg/kg). After 1 h, MSU crystals (2 mg/0.1 mL of PBS/mouse) or PBS alone were subcutaneously injected into the hind footpad of each mouse. After 24 h, the foot tissues were collected for analysis. (**A**) Time course of foot thickness changes. (**B**) MPO activity in the foot tissues was analyzed for neutrophil infiltration. (**C**) Immunoblotting of foot tissues for pro-caspase-1, caspase-1 (p20), and IL-1β expression. (**D**,**E**) ELISA for IL-1β and IL-18 expression in foot tissues. The values in (**A**,**B**,**D**,**E**) represent the means ± SEM (*n* = 5 mice); # significantly different from vehicle alone, *p* < 0.05; * significantly different from MSU alone, *p* < 0.05.

Next, we investigated whether loganin could prevent gout inflammation induced by NLRP3 inflammasome activation in a mouse foot gout model. Mice were orally administered loganin (5 or 30 mg/kg) or colchicine (1 mg/kg). One hour later, MSU crystals were injected into the hind foot plantar of the mice. MSU crystals injection resulted in increased foot thickness, while oral administration of loganin prevented foot thickness increase induced by MSU crystals injection (Figure 3A). Consistently, oral administration of loganin blocked MPO activity in foot tissues, which was increased by MSU crystals injection, showing that loganin reduced MSU crystals-induced neutrophil infiltration into foot tissues (Figure 3B). Loganin exhibited its inhibitory activity to a similar extent as colchicine treatment. These results demonstrate that loganin alleviates acute gout inflammation induced by MSU crystals deposition.

Furthermore, MSU crystals injection induced the cleavage of pro-IL-1β to IL-1β and of pro-caspase-1 to caspase-1 (p20) in foot tissues, whereas oral administration of loganin blocked the production of mature IL-1β and caspase-1 (p20) induced by MSU crystals (Figure 3C). Similarly, ELISA results showed that oral administration of loganin reduced the production of IL-1β and IL-18, increased by MSU crystals in foot tissues (Figure 3D,E). These results indicate that oral administration of loganin blocked MSU crystals-induced activation of the NLRP3 inflammasome in foot tissues.

Therefore, oral administration of loganin prevents the inflammatory symptoms of gouty arthritis induced by MSU crystals deposition, mediated by the suppression of NLRP3 inflammasome activation.

### 2.4. Loganin Prevents Mitochondrial Stress Induced by MSU Crystals

**Figure 4:**
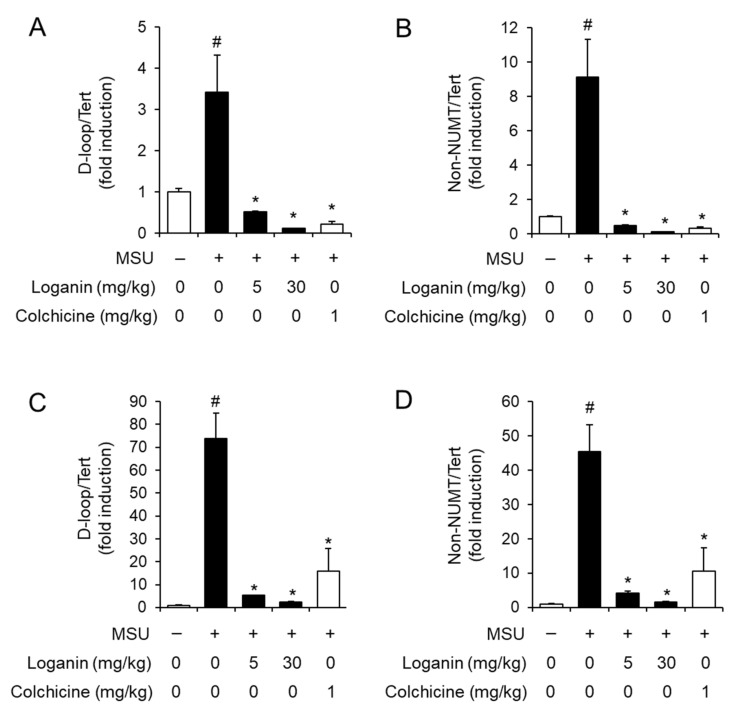
Oral administration of loganin suppresses mitochondrial DNA synthesis in gout tissues. The samples were obtained from the mice described in Figure 2 and Figure 3. The levels of mitochondrial DNA such as D-loop and non-nuclear mitochondrial DNA (NUMT) in (**A**,**B**) air pouch tissues and (**C**,**D**) foot tissues were quantified by qPCR analysis. The levels of nuclear DNA were measured to normalize mitochondrial DNA levels. The values represent the means ± SEM (*n* = 3); # significantly different from vehicle alone, *p* < 0.05; * significantly different from MSU alone, *p* < 0.05.

After being phagocytosed into macrophages, MSU crystals induce mitochondrial stress, which in turn leads to NLRP3 inflammasome activation [15]. Mitochondrial stress includes the synthesis of mitochondrial DNA (mtDNA), the loss of mitochondrial membrane potential (MMP, ΔΨm), and the production of mitochondrial reactive oxygen species (ROS) [15,16]. To investigate the mechanism by which loganin suppresses NLRP3 inflammasome activation, we examined whether loganin affected de novo synthesis of mtDNA in acute gout induced by MSU crystals deposition. Injection of MSU crystals increased the levels of mtDNA such as D-loop and non-nuclear mitochondrial DNA (NUMT) in air pouch tissues, while oral administration of loganin decreased the expression of D-loop and non-NUMT induced by MSU crystals (Figure 4A,B). Consistently, oral administration of loganin suppressed the expression of D-loop and non-NUMT induced by MSU crystals in foot tissues (Figure 4C,D). These results indicated that loganin suppressed de novo synthesis of mtDNA in MSU crystals-stimulated gout tissues, further suggesting that loganin prevents mitochondrial stress induced by MSU crystals.

**Figure 5:**
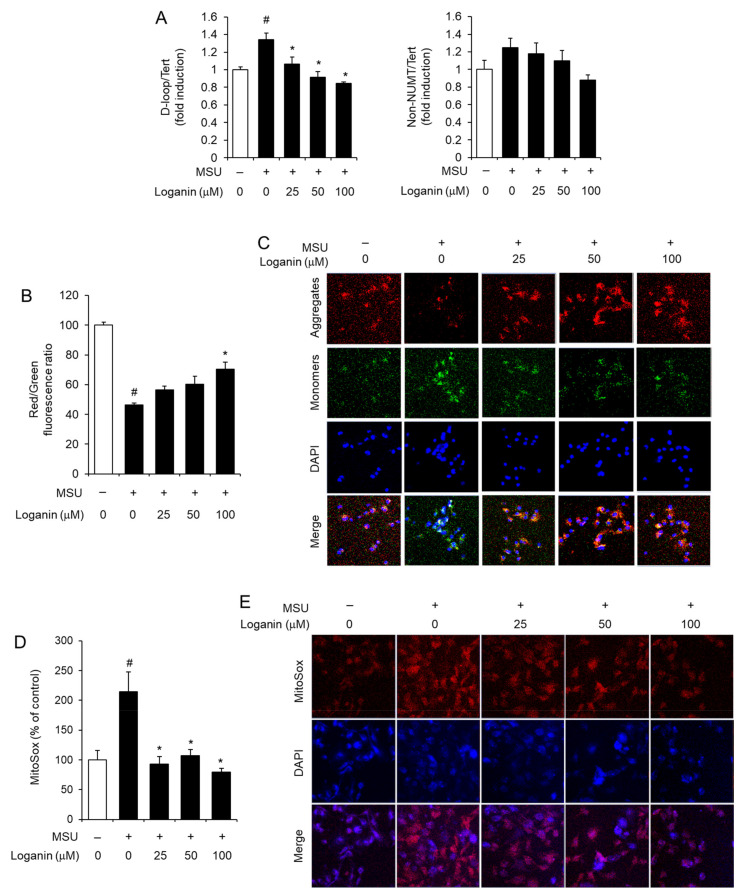
Loganin prevents mitochondrial dysfunction induced by MSU crystals in primary mouse macrophages. After LPS priming, BMDMs were treated with loganin for 1 h. Then, the cells were further stimulated with MSU crystals (500 μg/mL) for (**A**) 6 h or (**B**–**E**) 2 h. (**A**) The levels of mitochondrial DNAs such as D-loop and non-NUMT were determined by qPCR analysis. (**B**,**C**) For the measurement of mitochondrial membrane potential, BMDMs were stained with JC-1 (2 μM). (**B**) Red/green fluorescence ratios were determined with a fluorescence plate reader or (**C**) fluorescent images were obtained by confocal microscopy (magnification ×400). DAPI was used for nuclei staining. (**D**,**E**) For the measurement of mitochondrial reactive oxygen species, BMDMs were loaded with MitoSOX-Red (5 μM). (**D**) Fluorescence was determined by a fluorescence plate reader, or (**E**) fluorescent images were obtained by confocal microscopy (magnification ×400). The values in (**A**,**B**,**D**) represent the means ± SEM (*n*= 3); # significantly different from vehicle alone, *p* < 0.05; * significantly different from MSU alone, *p* < 0.05.

To further investigate the preventive effects of loganin on mitochondrial stress induced by MSU crystals, LPS-primed BMDMs were treated with loganin and subsequently stimulated with MSU crystals. Mitochondrial stress was determined by measuring the synthesis of mtDNA, the loss of MMP (ΔΨm), and the production of mitochondrial ROS. Loganin reduced the levels of mtDNA such as D-loop, which were increased by MSU crystals in macrophages (Figure 5A). MSU crystals treatment lowered MMP in macrophages, while loganin prevented MMP reduction as determined by JC-1 aggregate/monomer (red/green) fluorescence ratio (Figure 5B). Confocal imaging analysis showed consistently that loganin prevented the reduction of MMP induced by MSU crystals (Figure 5C). Furthermore, loganin decreased the production of mitochondrial ROS, which was increased by MSU crystals in macrophages, as measured by MitoSox fluorescence (Figure 5D). Confocal imaging analysis confirmed that loganin decreased mitochondrial ROS production induced by MSU crystals (Figure 5E).

The results demonstrated that loganin prevents mitochondrial stress induced by MSU crystals in macrophages. This suggests that loganin suppresses MSU crystals-induced NLRP3 inflammasome activation by blocking mitochondrial damage, culminating in the alleviation of acute gout inflammation derived from MSU crystal deposition.

## 3. Discussion

Gout is a type of inflammatory arthritis that is induced by the accumulation of MSU crystals in joints. MSU crystals induce the activation of the NLRP3 inflammasome and subsequent release of mature IL-1β, contributing to the development and progress of gouty arthritis. Our results show that oral administration of loganin prevented gout inflammation induced by MSU crystals via inhibition of NLRP3 inflammasome activation. Loganin reduced the production of mature IL-1β and IL-18 in macrophages and gout tissues stimulated with MSU crystals. The inhibitory effects of loganin on NLRP3 inflammasome activation were consistently observed in both cultured cell system and in vivo animal models.

MSU crystals are phagocytosed by cells, causing lysosomal damage that activates the NLRP3 inflammasome [13]. Lysosomal damage increases extracellular K^+^ levels, cathepsin B release, and production of ROS, which induce NLRP3 inflammasome activation [13]. While lysosomal damage is the prerequisite for MSU crystals-induced NLRP3 inflammasome, the precise mechanism linking lysosome rupture to NLRP3 inflammasome activation has not been completely understood. Mitochondrial dysfunction has been proposed to play a critical role in NLRP3 inflammasome activation in response to LPS and ATP [15]. Mitochondrial dysfunction is associated with generation of mitochondrial ROS, decrease in mitochondrial membrane potential, and release of mitochondrial DNA. De novo synthesis of mitochondrial DNA induced by a priming step promotes NLRP3 inflammasome activation [16]. Our results show that MSU crystals increased the levels of mitochondrial DNA in gouty tissues of mice. Similarly, MSU crystals decreased mitochondrial membrane potential and increased mitochondrial ROS levels in macrophages. These indicate that MSU crystals induce mitochondrial stress in macrophages and inflamed gouty tissues, suggesting the involvement of mitochondria dysfunction and consequent NLRP3 inflammasome activation in the pathogenesis of gout. In contrast, loganin exhibited protective effects on mitochondrial dysfunction by increasing mitochondrial membrane potential and decreasing mitochondrial ROS levels in macrophages. In addition, loganin reduced the levels of de novo synthesized mitochondrial DNA in both macrophages and gouty tissues. A recent study shows that ATP induces mitochondrial dysfunction, leading to cytosolic release of oxidized mitochondrial DNA to bind to and to activate the NLRP3 inflammasome [17]. Disruption of autophagy induces an increase of dysfunctional mitochondria and the cytosolic release of mitochondrial DNA in response to LPS and ATP in macrophages [15]. This suggests that loganin may regulate the upstream event of mitochondrial dysfunction such as autophagy, resulting in blockade of NLRP3 inflammasome activation. The effects of loganin on autophagy need to be elucidated in future studies.

Many phytochemicals and small-molecule inhibitors of the NLRP3 inflammasome have been reported [10]. We intend to search for new small molecule inhibitors with different inhibitory mechanisms and targets to provide eventually more effective strategies for drug development. Consistently with the protective effects of loganin on mitochondrial dysfunction, epigallocatechin-3-gallate (EGCG) suppressed the de novo synthesis of mitochondrial DNA, which was correlated with its inhibitory effects on NLRP3 inflammasome activation [18]. This suggests that inhibition of mitochondrial stress by small-molecule inhibitors could be an efficient strategy to suppress the activation of the NLRP3 inflammasome. Mitochondrial dysfunction in connection with NLRP3 inflammasome activation has been reported to play a role in inflammatory diseases such as diabetes, atherosclerosis, neurological disorders, cardiovascular disease, and kidney disease [19,20]. Therefore, the beneficial effects of loganin on mitochondrial dysfunction could be applied to other NLRP3-related diseases.

In general, effective concentrations and sensitivity of chemicals could be variable in different experimental conditions, depending, for example, on cell type, treatment time, stimulators, and biomarkers. Cheng et al. showed the inhibitory effects of loganin at 1 μM on high-glucose-induced NLRP3 inflammasome activation [21]. Cheng et al. performed these experiments in a rat Schwann cell line treating with loganin for 48 h, while we used primary macrophages isolated from mouse bone marrow and treated with loganin for maximum 7 h. Cheng et al. used a high glucose concentration to induce NLRP3 inflammasome activation, whereas we used MSU crystals as an NLRP3 activator. Moreover, Cheng et al. determined the inhibitory effects of loganin on the priming step involving NF-κB activation induced by high glucose levels and showed that loganin decreased the mRNA expression of *Nlrp3*, *Asc*, *Caspase-1*, and *Il-1β*, which were upregulated upon NF-κB activation. In contrast, we intended to investigate the effect of loganin on the activation step induced by MSU crystals, not on the priming step induced by LPS, by adding loganin after washing out LPS. Although we used loganin up to 100 μM, its inhibitory effects began at a concentration of 25 μM, as shown in Figure 1B,C, and Figure 5A,D and were evident at a concentration of 50 μM. Other studies used similar concentration ranges of loganin, as loganin reduced the production of pro-inflammatory cytokines, TNF-α, IL-6, and monocyte chemoattractant protein-1 (MCP-1) and the activation of NF-κB in apoCIII-stimulated mouse 3T3L1 adipocytes at 16 μM–128 μM [14]. Loganin at 100 μM was effective to inhibit rat renal mesangial cell proliferation induced by advanced glycation end products [22].

Since gout is a recurrent inflammatory disease, gout patients suffer relapsing gout flares, despite currently used medications are effective to reduce inflammation and pain. Thus, it is necessary to develop more effective therapeutic strategies for preventing gout inflammation and relapsing gout flares. Inhibitors of IL-1 such as anakinra, rilonacept, and canakinumab have been investigated. However, their use is limited by their high cost, inconvenient treatment routes, and side effects [23,24]. Therefore, an effective oral administration of loganin would confer additional advantages, including accessibility, cost, and convenience. To the best of our knowledge, the current in vivo experimental gout models induce an acute gout attack by injecting MSU crystals into air pouches, footpads, ankle joints, or knee joints. Accordingly, we used two acute gout models, an air pouch model and a footpad gout model to study the effects of loganin on gout inflammation. Nevertheless, information on the long-term effects of loganin would be valuable to evaluate the application of loganin for the clinical treatment of gout. Since gout is characterized by recurrent attacks, a long-term model of gout needs to be established, so that the effectiveness of treatments could be evaluated for relapsing gout attacks. The long-term effects of loganin on recurrent gout attacks need to be further investigated in future studies.

## 4. Materials and Methods

### 4.1. Animals

C57BL/6 mice (male) were obtained from Raon Bio (Seoul, Korea) and were acclimated in specific pathogen-free conditions in an animal facility for at least one week before experimentation. The mice were housed in a room controlled for optimal temperature (23 ± 3 °C) and relative humidity (40–60%) under specific pathogen-free condition. Animal care and the experimental protocols were carried out in accordance with the guidelines of the Institutional Animal Care and Use Committee (IACUC) of the Catholic University of Korea (permission #, 2014-006, 2017-015). Mice of individual experimental groups in each experiment were of similar age and weight and were randomly allocated to treatment groups. Investigators were blinded for the treatment of mice in all experiments.

### 4.2. Cell Culture

BMDMs were prepared after bone marrow was isolated from C57BL/6 mice as described previously [25]. Macrophages were cultured in Dulbecco’s modified eagle medium containing 10% (*v*/*v*) fetal bovine serum (Corning, Somervile, MA, USA), 10,000 units/mL penicillin, and 10,000 μg/mL streptomycin (Thermo Fisher Scientific, Waltham, MA, USA).

### 4.3. Reagents

Purified lipopolysaccharides (LPS) from *Escherichia coli* were obtained from List Biological Laboratory (Santa Clara, CA, USA) and dissolved in endotoxin-free water. Loganin (purity: ≥97.0%) was purchased from Sigma-Aldrich (St. Louis, MI, USA). Monosodium urate crystals were purchased from Invivogen (San Diego, CA, USA). The antibody for caspase-1 was obtained from Santa Cruz Biotechnology (Dallas, TX, USA). The antibody for IL-1β was obtained from R&D Systems (Minneapolis, MN, USA).

### 4.4. Analysis of Inflammasome Activation

BMDMs were seeded at 2 × 10^6^ cells/well in 6-well plates for immunoblot assays and 4 × 10^5^ cells/well in 96-well plates for ELISAs. BMDMs were primed with LPS for 4 h. To exclude the effect of loganin on the LPS priming step, loganin was added after washing out LPS with phosphate-buffered saline (PBS). After treatment with loganin, the cells were further stimulated with MSU crystals in a serum-free medium. The cell supernatants and lysates were processed for immunoblot assays as previously described [26].

### 4.5. Enzyme-Linked Immunosorbent Assays

IL-1β levels were determined using a DuoSet enzyme-linked immunosorbent assay (ELISA) kit (R&D system, Minneapolis, MN, USA) according to the manufacturer’s instructions. IL-18 levels were determined using an ELISA kit (Invitrogen, Carlsbad, CA, USA) according to the manufacturer’s instructions.

### 4.6. Confocal Microscopy Analysis for ASC Specks

This was performed as described previously [27]. Briefly, BMDMs were fixed with methanol for 30 min, blocked with 1% bovine serum albumin for 30 min, and incubated with an anti-ASC antibody (Santa Cruz Biotechnology Inc., Dallas, TX, USA) at 4 °C overnight. The cells were further incubated with an anti-rabbit IgG-FITC antibody (Invitrogen, Carlsbad, CA, USA) and co-stained with 4′,6-diamidino-2-phenylindole (DAPI, 1 μg/mL; Invitrogen) for nuclei visualization. Samples were examined with an LSM710 laser scanning confocal microscope (Carl Zeiss, Oberkochen, Germany). Images were obtained and analyzed with ZEN2011 software (Carl Zeiss, Oberkochen, Germany).

### 4.7. Cell Viability Assay

Cell viability was analyzed by MTT (3-(4,5-dimethylthiazol-2-yl)-2,5-diphenyltetrazolium bromide) assay. After BMDMs were treated with loganin for 7 h, MTT (5 mg/mL; Cas# 298-93-1, Merck, Kenilworth, NJ, USA) was added to each well and incubated at 37 °C for 4 h. Media was removed and cells were dissolved in DMSO. The optical density was measured at 540 nm using a plate reader (MAGPIX®, Luminex Corporation, Austin, TX, USA).

### 4.8. Air Pouch Inflammation Model

An air pouch was formed in the back of C57BL/6 mice (8 weeks old) by subcutaneous injection of sterile air, as previously described [28]. On day 4, loganin (5 or 30 mg/kg), colchicine (1 mg/kg), or vehicle (0.02% DMSO) in 0.2 mL sterilized water was orally administered to mice. After 1 h, MSU crystals (3 mg/1 mL of sterile, endotoxin-free PBS) or PBS were injected into the pouch. Six hours later, the mice were anesthetized, and the air pouch lavages were collected with 2 mL PBS containing 5 mM EDTA and processed for ELISAs, immunoblot assays, and determination of MPO activity.

### 4.9. Mouse Foot Gout Model

C57BL/6 mice (8 weeks old) were orally administered loganin (5 or 30 mg/kg) colchicine (1 mg/kg), or vehicle (0.02% DMSO) in 0.2 mL sterilized water. After 1 h, MSU crystals (2 mg/0.1 mL of sterile, endotoxin-free PBS) or PBS were administered via subcutaneous injection on plantar surface of the hind foot [28]. Changes in foot thickness were measured for 24 h. The foot tissues were homogenized in RIPA buffer (50 mM Tris-HCl, pH 7.4, 1% NP-40, 0.25% sodium deoxycholate, 150 mM NaCl, 1 mM EGTA, 1 mM PMSF, 1 mM Na_3_VO_4_, 10 μg/mL leupeptin), and the homogenates were centrifuged at 12,000× *g* for 10 min. The supernatants were used for MPO assays, ELISAs, and immunoblot assays.

### 4.10. Myeloperoxidase Activity Assay

The air pouch lavages and the supernatants of foot tissue homogenates were prepared as described in Section 4.8 and Section 4.9. MPO, a representative marker for the activation and infiltration of neutrophils, was measured with a MPO Colorimetric activity assay kit (Catalog# K744, Bio Vision, Milpitas, CA, USA) according to the manufacturer’s protocol.

### 4.11. Quantitative Real-Time Polymerase Chain Reaction Analysis

Total DNA was isolated with the G-spin^TM^ DNA Mini Kit (iNtRON Biotechnology, Seongnam, Korea) according to the manufacturer’s instruction. Quantitative real-time polymerase chain reaction was performed as previously described [18]. Mitochondrial DNA was quantified by qPCR using primers specific for the mitochondrial D-loop region or not inserted in nuclear DNA (non-NUMT). Nuclear DNA encoding *Tert* was used for normalization. Primer sequences were as follows: D-loop F: 5′-AATCTACCATCCTCCGTGAAACC-3′, D-loop R: 5′-TCAGTTTAGCTACCCCCAAGTTTAA-3′; non-NUMT F: 5′-CTAGAAACCCCGAAACCAAA-3′, non-NUMT R: 5′-CCAGCTATCACCAAGCTCGT-3′; *Tert* F: 5′-CTAGCTCATGTGTCAAGACCCTCTT-3′, *Tert* R: 5′-GCCAGCACGTTTCTCTCGTT-3′. Specificity of the amplified PCR products was assessed by melting curve analysis.

### 4.12. Measurement of Mitochondrial Membrane Potential 

The MitoProbe^TM^ JC-1 assay kit (Thermo Fisher Scientific, Waltham, MA, USA) was used for the measurement of mitochondrial membrane potential. After LPS-primed BMDMs were treated with loganin, the cells were further stimulated with MSU crystals in serum-free medium. After incubating the JC-1 probe with the cells at 37 °C for 30 min, the cells were washed twice with PBS. Fluorescence intensity was measured at 514/529 (red/green) and 590 nm with a plate reader (Synergy H1, Biotek, Winooski, VT, USA). To obtain fluorescent images by confocal microscopy, after cell incubation with JC-1 at 37 °C for 30 min, the cells were washed and mounted on glass slides. JC-1 monomers were detected by green fluorescence, while JC-1 aggregates were detected by red fluorescence. Images were analyzed by an LSM710 laser scanning confocal microscope (Carl Zeiss, Oberkochen, Germany) with ZEN2011 software (Carl Zeiss).

### 4.13. Determination of Mitochondrial Reactive Oxygen Species

The measurement of mitochondrial reactive oxygen species was performed using MitoSOX^TM^ (Thermo Fisher Scientific, Waltham, MA, USA). After LPS-primed BMDMs were treated with loganin, the cells were further stimulated with MSU crystals. The cells were incubated with MitoSOX-Red (5 μM) at 37 °C for 30 min and washed gently with PBS. MitoSOX-Red fluorescence was measured at 580 nm after excitation at 510 nm, with a plate reader (Synergy H1, Biotek, Winooski, VT, USA). For confocal microscopy imaging, the cells were incubated with MitoSOX-Red and DAPI. After incubation for 30 min, the cells were washed with PBS and mounted with mounting solution (VECTASHIELD Antifade Mounting Medium, LSBio, Seattle, WA, USA). Confocal Microscopy images were analyzed with ZEN2011 software (Carl Zeiss) by LSM710 laser scanning confocal microscope (Carl Zeiss, Oberkochen, Germany).

### 4.14. Statistical Analysis

Statistical analysis was performed by the software GraphPad Prism 5 (GraphPad Software, San Diego, CA, USA). All data were analyzed by one-way ANOVA followed by Tukey’s multiple comparison test and expressed as means ± SEM. Values of *p* < 0.05 were considered significant. The graphs or pictures are representative of at least two or three independent experiments.

## 5. Conclusions

The results of this study identified loganin as an inhibitor of NLRP3 inflammasome activation induced by MSU crystals in mouse primary macrophages and in vivo mouse acute gout models. Loganin inhibited MSU crystals-induced secretion of inflammatory cytokines such as IL-1β and IL-18, resulting in the suppression of gout inflammation in tissues injected with MSU crystals. This work demonstrates that the prevention of mitochondrial stress can be a relevant mechanism through which loganin mediates NLRP3 inhibition. This finding shows that the administration of loganin could be an efficient prevention strategy for acute gouty arthritis, further suggesting the possibility to use loganin to prevent other inflammatory diseases related to NLRP3 inflammasome activation.

## Data Availability

The data presented in this study are available in article or supplementary material here.

## Supplementary Materials

The Supplementary Materials are available online. Figure S1: Loganin does not inhibit the production of IL-6 and TNF-α in primary macrophages.

## Sample Availability

Samples of the compounds are not available from the authors.
