# Supplementary material for: Loganin Alleviates Gout Inflammation by Suppressing NLRP3 Inflammasome Activation and Mitochondrial Damage
Source: Molecules. 2021 Feb 18;26(4):1071. doi: 10.3390/molecules26041071 (PMC7923023; doi:10.3390/molecules26041071)
Supplement: Supplementary file 1 [file molecules-26-01071-s001.pdf]

Supplementary Material

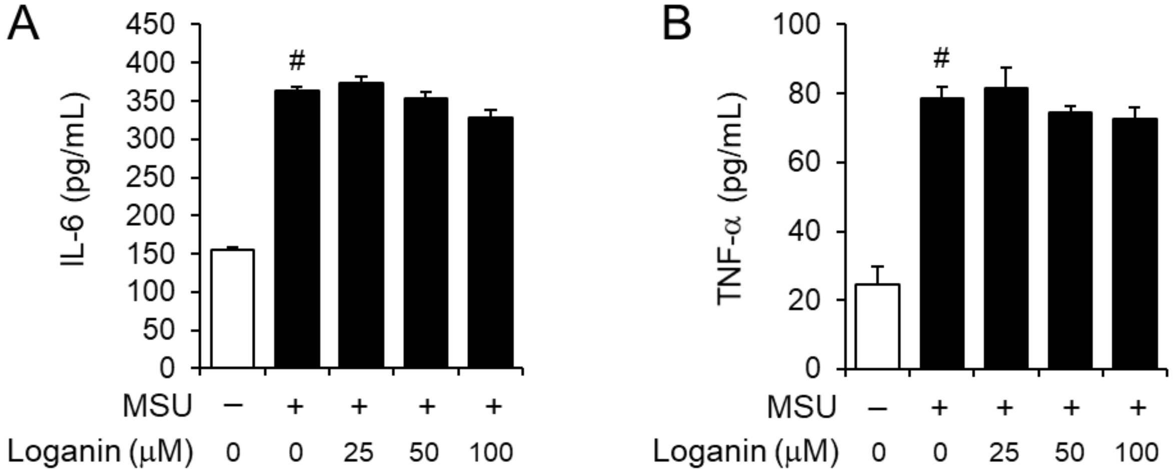

Figure S1. Loganin does not inhibit the production of IL-6 and TNF-α in primary macrophages. (A, B) Bone marrow-derived mouse primary macrophages (BMDMs) were primed with LPS (100 ng/mL) for 4 h. The cells were treated with loganin for 1 h and then stimulated with MSU crystals (500 μg/mL) for 4.5 h. Cell culture supernatants were analyzed for secreted IL-6 and TNF-α by ELISA. The values represent the means ± SEM (A, $n = 8$; B, $n = 3$). # Significantly different from vehicle alone, $p < 0.05$.
